# *Duhuo Jisheng Tang *for treating osteoarthritis of the knee: a prospective clinical observation

**DOI:** 10.1186/1749-8546-2-4

**Published:** 2007-03-30

**Authors:** Jung-Nien Lai, Huey-Jeng Chen, Chao-Chung Chen, Jer-Huei Lin, Jing-Shiang Hwang, Jung-Der Wang

**Affiliations:** 1 Department of Obstetrics and Gynecology; Department of Chinese Medicine, Taipei City Hospital, Yangming Branch; Institute of Traditional Medicine, School of Medicine, National Yang-Ming University, No.155, Sec. 2, Linong Road, Taipei 112, Taiwan; 2 Division of Chinese Internal medicine, Taipei City Hospital, Branch for Chinese Medicine, No.100, Kunming St., Taipei 108, Taiwan; 3 Department of Traumatology, Taipei City Hospital, Branch for Chinese Medicine, No.100, Kunming St., Taipei 108, Taiwan; 4 Division of Pharmacognosy, Bureau of Food and Drug Analysis, Department of Health, Executive Yuan, No.161-2, Kunyang St., Taipei 115, Taiwan; 5 Institute of Statistical Science, Academia Sinica, No.128, Academia Road Sec. 2, Taipei 115, Taiwan; 6 Departments of Internal Medicine and Environmental and Occupational Medicine, National Taiwan University Hospital and Institute of Occupational Medicine and Industrial Hygiene, National Taiwan University College of Public Health, No.17, Xuzhou Road, Taipei 100, Taiwan

## Abstract

**Background:**

Little scientific evidence supports the efficacy of herbal medicines in the treatment of degenerative arthritis of the knee. The purpose of this study is to evaluate both the efficacy and safety of a finished Chinese herbal preparation *Duhuo Jisheng Tang (DJT) *in reducing symptoms of degenerative osteoarthritis of the knee.

**Methods:**

A prospective follow-up study was carried out in two hospitals in Taipei between April and October 2005. Sixty-eight osteoarthritis patients, with symptoms diagnosed by radiologists, received *DJT *at a rate of 2.5 g, twice daily for four weeks. Baseline scores were measured on the Western Ontario and McMaster Universities Osteoarthritis (WOMAC) index, followed by further measures at the end of weeks 1, 2 and 4. The World Health Organization Quality of Life (WHOQOL) assessment was undertaken as a secondary outcome, with pattern identification questionnaires being adopted. Regression models were constructed to explore the score differences between the baseline and at weeks 2 and 4 by various determinants including age, gender, body mass index (BMI), severity at baseline, use of rescue medication, aversion to cold and flaccidity of the lower back and knees.

**Results:**

Among the 68 participants, there were statistically significant reductions in the WOMAC index scores for pain, stiffness and physical functioning in the second and fourth weeks, with effects first appearing during week 2. By week 4, the mean WOMAC index scores had fallen from 22.2 (± 19.2) to 16.1 (± 16.2) for pain, from 28.1 (± 24.9) to 18.5 (± 20.3) for stiffness, and from 22.6 (± 18.0) to 18.2 (± 17.8) for physical functioning, while the global score for pain under the visual analogue scale (VAS) was reduced from 38.7 (± 21.5) to 27.8 (± 19.8).

**Conclusion:**

In the treatment of degenerative osteoarthritis of the knee, a 4-week therapy with the Chinese herbal preparation *DJT *reduced pain and stiffness and improved physical functioning, but it was less effective in treating flaccidity and aversion to cold.

## Background

Osteoarthritis (OA) is the most common form of joint disease, with the most common location being the knee [1]. As the patient ages, or the illness worsens, OA becomes associated with incapacity and deterioration in the quality of life owing to increased pain and loss of mobility [2]. For those OA patients who are not suited for surgery and/or those with ineffective medical treatment and/or intolerable side effects [3], complementary and alternative management approaches are frequently considered.

According to traditional Chinese medicine, arthritis belongs to the category of *bi*-syndrome, which is attributed to the weakening of the body's protective energy (*weiqi*). Weakened *qi *allows the incursion of the external pathogens (*waixie*) such as 'Wind', 'Cold' and 'Dampness' in Chinese medicine terminology. This incursion impedes the normal flow of *qi *and results in pain and abnormal function of the human body. A combination of symptoms such as pain, stiffness, flaccidity and aversion to cold of the knee signifies disharmony caused by 'Wind', 'Cold' and 'Dampness'.

First documented about thirteen centuries ago in *Qianjin Yaofang *(*Invaluable Prescriptions for Ready Reference*), an ancient Chinese medicinal text [4,5], *Duhuo Jisheng Tang (DJT) *was used to treat symptoms caused by 'Wind', 'Cold' and 'Dampness'. *DJT *is a mixture of fifteen plant species as follows: *Radix Angelicae Sinensis (Danggui)*, *Radix Paeoniae Alba(Baishao)*, *Radix et Rhizoma Glycyrrhizae (Gancao)*, *Radix Rehmanniae (Dihuang)*, *Radix et Rhizoma Ginseng (Renshen)*, *Poria (Fuling)*, *Radix Angelicae Pubescentis (Duhuo)*, *Herba Taxilli (Sangjisheng)*, *Radix Gentianae Macrophyllae (Qinjiao)*, *Radix Saposhnikoviae (Fangfeng)*, *Radix et Rhizoma Asari (Xixin)*, *Rhizoma Chuanxiong (Chuanxiong)*, *Cortex Cinnamomi (Rougui)*, *Cortex Eucommiae (Duzhong)*, *Radix Cyathulae (Chuanniuxi) *[5] (Table 1).

**Table 1:** Nomenclature of Chinese herbs used in *Duhuo Jisheng Tang*

**Pharmaceutical name**	**Chinese pinyin**	**Latin botanical name**
*Radix Angelicae Sinensis*	*Danggui*	*Angelica sinensis (Oliv.) Diels*
*Radix Paeoniae Alba*	*Baishao*	*Paeonia lactiflora Pall*
*Radix et Rhizoma Glycyrrhizae*	*Gancao*	*Glycyrrhiza uralensis Fisch*.
*Radix Rehmanniae*	*Dihuang*	*Rehmannia glutinosa Libosch*.
*Radix et Rhizoma Ginseng*	*Renshen*	*Panax ginseng C. A. Mey*.
*Poria*	*Fuling*	*Poria cocos (Schw.) Wolf*
*Radix Angelicae Pubescentis*	*Duhuo*	*Angelica pubescens Maxim.f. biserrata Shan et Yuan*
*Herba Taxilli*	*Sangjisheng*	*Taxillus chinensis (DC.) Danser*
*Radix Gentianae Macrophyllae*	*Qinjiao*	*Gentiana macrophylla Pall*.
*Radix Saposhnikoviae*	*Fangfeng*	*Saposhnikovia divaricata (Turcz.) Schischk*.
*Radix et Rhizoma Asari*	*Xixin*	*Asarum heterotropoides Fr. Schmidt var. mandshuricum (Maxim.) Kitag*.
*Rhizoma Chuanxiong*	*Chuanxiong*	*Ligusticum chuanxiong Hort*.
*Cortex Cinnamomi*	*Rougui*	*Cinnamomum cassia Presl*
*Cortex Eucommiae*	*Duzhong*	*Eucommia ulmoides Oliv*.
*Radix Cyathulae*	*Chuanniuxi*	*Cyathula officinalis Kuan*

Aristolochic acid I (AA-I), a known nephrotoxin [6], is found in a commonly used Chinese medicinal herb *Xixin *which is originated from nine Asarum species (*Aristolochiaceae*). All products containing *Xixin *have been prohibited in the US and Canada [7,8]. However, *Xixin *remains widely used in the *DJT *formula in Korea, Japan, Mainland China and Taiwan. A previous study showed that the side effects of *DJT *appeared at a daily dose of 9.0 g [11]. The commonly administered dosage in Taiwan is 5 g per day [9]. In 2003, the Committee of Chinese Medicine and Pharmacy (CCMP) in Taiwan removed all Aristolochic acid containing herbs except *Xixin *from the approved herbal products because of safety concerns. The committee also stipulated that the AA-I level in all *Xixin *preparations should be undetectable [10]. The current study is intended to evaluate the efficacy of *DJT *on patients with OA of the knee at a daily dose of 5.0 g. The observation period was to last four weeks to test whether there are any cumulative effects. We also intended to verify an ancient indication that *DJT *was effective in treating pain, stiffness, flaccidity of the lower back and knee and aversion to cold.

## Methods

The herbal preparation *DJT *used in this study was prepared under GMP (Good Manufacturing Practices) and provided by Sun Ten Pharmaceutical (Taiwan). The project was entirely funded by the CCMP to detect potential toxicity or adverse effects of *DJT*. Apart from providing the batch of *DJT*, Sun Ten Pharmaceutical was not involved in any other sponsorship, study design or monitoring of the participants.

### Preparation of the herbs

All herbal components of *DJT *were prepared in a large computer-controlled boiler where volatile oils of therapeutic values were collected and sprayed into a two-story high vacuum drying chamber. The active ingredients of the herbs were transformed into granulated compounds five times more concentrated than the raw herbs. These granulated compounds were then vacuum dried at low temperatures before being siphoned into a separate sterile-room where they were bottled, labeled and sealed. For quality assurance of the active ingredients in *DJT*, HPLC (high performance liquid chromatography) fingerprinting was employed to identify substances in the final product [12]. An extraction process required by the CCMP regulations was performed, involving the use of water to eliminate AA-I from *Asarum heteropoides *roots, a nephrotoxic substance contained mostly in the leaves and/or aerial parts of plants [13]. Liquid chromatography – tandem mass spectrometry (LC/MS/MS) [14], a more sensitive method, had been carried out by the Bureau of Food and Drug Analysis of the Department of Health prior current study to ensure that AA-I was at an undetectable level. Each batch of *DJT *was also tested for *E. coli, Salmonella *(bacteria count) and heavy metals.

### Recruitment of subjects

This prospective follow-up study was coordinated from the National Taiwan University, while participants were enrolled through two research clinics in northern Taiwan: Chinese Medicine Branch and Yangming Branch of the Taipei City Hospital. Approval of the study was obtained from the Joint Institutional Review Board for Traditional Chinese Medicine of Taiwan (JIRBTCM94-0426-01). All participants provided signed and informed consents before taking part in the study.

#### Inclusion criteria

Participants were recruited through newspaper advertisements and flyers posted in clinics and health fairs between April and October 2005. The status of osteoarthritis of the knee was confirmed by radiography (mild to moderate osteophytes and/or joint space narrowing). Qualified participants were at least 18 years of age with symptomatic osteoarthritis in at least one knee and had sought medical help over the two-week period prior to the study. Participants had not been involved in any other medical trials three months prior to the study. Participants were required to discontinue the use of any current medications, including any conventional or herbal products for arthritis, at least two weeks prior to the initial screening for the study.

#### Exclusion criteria

Rheumatoid, inflammatory or any other type of arthritis; arthroscopy or intra-articular corticosteroids/hyaluronic acid injections in the previous month; any evidence of renal or liver dysfunction as defined by a level of at least 1.5 times the upper reference limit [serum creatinine: 1.3 mg/dl, blood urea nitrogen (BUN): 22 mg/dl, serum aspartate-aminotransferase (AST): 25 IU/L, alanine-aminotransferase (ALT): 29 IU/L]; uncontrolled hypertension; diabetes mellitus; or any signs of cancer.

### Study design and procedure

All nurses involved in the study attended a standardized training session to ensure consistency and to meet GCP (Good Clinical Practices) requirements in the study protocol. Participant eligibility was assessed during the first two visits to the clinic. Following the initial screening visit, participants entered a run-in phase to determine baseline data on their symptoms and their quality of life; each participant also underwent a health assessment including complete blood counts and biochemical function tests. These data were collected to ensure the eligibility criteria for each participant, and to screen out respondents with potential poor compliance. Following the run-in phase, each participant was provided with sufficient *DJT *to begin treatment at a dose of 2.5 g twice daily.

Subsequent study visits were scheduled for the first, second and fourth weeks, with any symptoms of the knee and adverse events being assessed at each visit. At the end of the trial, the participants received a further physical examination, including blood tests. Participants were contacted by telephone one to two days prior to each visit in order to encourage their continued compliance. Throughout the study period, leftover package counts were undertaken so as to monitor each participant's compliance. The consumption of non-steroidal anti-inflammatory drugs (NSAIDs) rescue medication was also recorded at each visit.

### Efficacy/Tolerability

The primary outcome parameters evaluated prior to and during the four-week intervention were pain, stiffness and physical functioning subscales, along with the global pain assessment using a 100-mm 'visual analog scale' (VAS) from the Western Ontario and McMaster Universities Osteoarthritis (WOMAC) index [15]. We purchased the WOMAC Chinese language form directly from the author [16]. Changes in the quality of life were also assessed using the World Health Organization Quality of Life questionnaire – Taiwan brief version (WHOQOL-BREF) [17]. Each subject's physical constitution was also categorized using a questionnaire for pattern identification, or '*bian zheng*' in Chinese medical terminology, based on the criteria recommended in *Zhongyao Xinyao Linchuang Yanjiu Zhidao Yuanze (The Guidelines for Clinical Research on New Chinese Medicines) *[18]. The questionnaire consisted of categories as follows: frequency of pain, aversion to cold and flaccidity of the lower back and knees (each of which were assessed in terms of 'occasional or less', 'often', or 'so frequent as to interfere with work'), and duration of stiffness ('less than one hour' or 'more than one hour').

The WOMAC index is a multi-dimensional, disease specific, self-administered, health status measure. It probes clinically important, patient-relevant symptoms in the areas of pain, stiffness and physical function in patients with knee osteoarthritis. The instrument comprises 24 questions (five on pain, two on stiffness and 17 on physical functioning) and can be completed in less than five minutes. Weightings were introduced to add together the five items on pain as a total sum score which predominantly quantifies the severity of knee pain in different daily activities, with the same process being carried out on stiffness and physical functioning [19]. The VAS version of the WOMAC is valid, reliable and sufficiently sensitive for the detection of clinically-important changes in health status following a variety of interventions [20,21]. The Taiwan version of the WHOQOL-BREF comprises four domains (i.e. physical, psychological, social and environmental) containing 24 facets, and two national items on overall quality of life (QOL) and general health [17], with higher scores indicating superior QOL.

### Safety assessment

Routine hematology and biochemistry data were collected at the baseline and at week 4, including complete blood count and platelets, serum levels of creatinine, BUN, AST, ALT, albumin/globulin (A/G), uric acid and urine levels of N-acetyl-β-D-glucosaminidase and retinol binding protein. A research nurse also actively monitored any adverse events and recorded any unexpected signs, symptoms or feelings during the study period.

### Statistical analysis

Our analysis focused mainly on changes in the three-domain construct of the WOMAC assessment (pain, stiffness and physical function) along with the WHOQOL measures. The treatment effects were described as changes of the mean differences among the participants between the scores measured during each visit at weeks 1, 2, and 4 and at baseline. Separate linear regression models were constructed for the response variables of the differentiated scores on the pain, stiffness and physical functioning subscales, the VAS-pain and the WHOQOL-BREF domains.

The explanatory variables in the final regression model were gender, age, baseline severity of osteoarthritis, the interaction between age and gender, the BMI (in kg/m^2^), aversion to cold, flaccidity of the lower back and knees and usage of NSAID rescue drugs. For us to examine the improvement across the 3 visits, two indicator variables for visits at weeks 2 and 4 were also included in the regression model. The coefficients of the two indicator variables represent the differences between the average value of measured scores for the visits at weeks 2 and 4 versus those at week 1. The error terms were assumed to be correlated among the three repeated measurements on each participant. Finally, each single WOMAC score item was also examined separately so as to identify any WOMAC items sensitive to treatment by *DJT*. The estimates of coefficients and standard errors of the estimates in the multiple regression models with correlated error terms were obtained by using generalized least squares function of the package nlme version 3.1–60 in a free statistical software R version 2.1.1 [22].

## Results

### Subjects

Of the 87 sample patients, 18 were deemed ineligible. The principal reasons for ineligibility were uninterested in participation (n = 9), abnormal liver function (n = 5), abnormal renal function (n = 2) and no evidence of osteoarthritis (n = 2). Sixty-eight of the initial 69 participants completed the four-week study. The main reason for the withdrawal was lack of efficacy during the first week of treatment. The demographic and clinical characteristics of the study subjects are summarized in Table 2. Among them, 37% were male, 82% married and 59% having attained senior high school education or above. Seven of the 68 took less than 80% of the prescribed dosage over the study period for the reason of lack of response to *DJT*. Patients with poor compliance had significantly lower scores in severity of pain and physical functioning and had significantly higher scores in physical QOL than those with good compliance.

**Table 2:** Baseline demographic and clinical characteristics for the 68 participants

**Definition**	**Mean (SD)**
Age (years)	59.2 (10.2)
Body mass index	24.0 (3.2)
Gender	
% Male	37
Religion	
% Buddhist	56
% Christian	10
Marital states	
% Married	82
% Divorced/Widowed/Separated	15
Education	
% Junior high or below	41
% University or above	34
Pain in visual analogue scale	38.7 (21.5)
WOMAC Scores^a^	
Pain	22.2 (19.2)
Stiffness	28.1 (24.9)
Physical functioning	22.6 (18.0)
Flaccidity of the lower back and knees	
% Mild	54
% Moderate	32
% Severe	13
Aversion to cold	
% Mild	73
% Moderate	12
% Severe	15
WHOQOL-BREF Scores^b^	
Physiological domain	12.9 (2.4)
Psychological domain	13.0 (2.2)
Social domain	13.5 (1.9)
Environment domain	13.5 (1.7)

^a ^WOMAC refers to the Western Ontario and McMaster Universities Osteoarthritis Index.

^b ^WHOQOL – BREF refers to the World Health Organization Quality of Life – brief version.

### Efficacy on pain, stiffness and physical functioning

After two weeks of treatment, there were significant and persistent improvements in each of the WOMAC subscales and also in the 10 cm VAS score for knee pain (Table 3). The magnitudes of the improvements in the WOMAC subscales were 27.5% for pain, 34.2% for stiffness and 19% for physical functioning. There was also a 28.2% improvement in the VAS score.

**Table 3:** Means and standard deviation of major variables measured at baseline and weeks 1, 2 and 4

**Variable definition**	**Baseline**	**Week 1**	**Week 2**	**Week 4**
WOMAC Scores^a^	Mean (SD)	Mean (SD)	Mean (SD)	Mean (SD)
Pain	22.2(19.2)	21.4(20.9)	16.9(16.9) **	16.1(16.2) **
Stiffness	28.1(24.9)	25.8(23.1)	17.4(18.5) ***	18.5(20.3) ***
Physical functioning	22.6(18.0)	22.4(19.5)	18.2(15.8) **	18.2(17.8) *
Pain in visual analogue scale	38.7(21.5)	33.0(20.7)*	27.0(21.5) ***	27.8(19.8) ***
WHOQOL-BREF Scores^b^	-	-	-	-
Physiological domain	12.9(2.4)	-	-	13.2(1.9)
Psychological domain	12.4(2.0)	-	-	12.3(1.5)
Social domain	13.5(1.9)	-	-	13.6(2.2)
Environment domain	13.5(1.7)	-	-	13.4(1.5)

^a ^WOMAC refers to the Western Ontario and McMaster Universities Osteoarthritis Index.

^b^WHOQOL – BREF refers to the World Health Organization Quality of Life – brief version.

* indicates *P *< 0.05; ** indicates *P *< 0.01; and *** indicates *P *< 0.001.

After four weeks of treatment, 44 of the 68 patients reported no change in the symptom of flaccidity; nine reported improvements, whereas fifteen reported deterioration. Thirty-eight patients reported no change in the symptom of aversion to cold after four weeks of treatment; fourteen reported improvements, while16 reported deterioration.

There were no significant improvements in these two symptoms after the treatment.

The results of the multiple linear regression analyses indicate the effects of the different determinants on the outcome scores of the VAS for pain and different WOMAC subscales (Table 4). After rescue medication and other determinants had been controlled, the model constructions demonstrated significant improvements in the severity of pain, stiffness and physical functioning after week 2 (which also persisted through week 4), with substantial treatment effects on the stratum of the most severe pain (baseline VAS score >4 cm, n = 32). The frequency of either flaccidity or aversion to cold were not significantly affected in any of the above major outcome scores, and there were no consistent improvements with regard to these two symptoms after week 4.

**Table 4:** The estimates of regression coefficients and standard errors for modeling outcomes of VAS for pain and WOMAC subscales

			**WOMAC subscales^b^**					
**Variable definition**	**VAS for pain^a^**							
			**Pain**		**Stiffness**		**Physical function**	
	**Coeff**.	**S.E**.	**Coeff**.	**S.E**.	**Coeff**.	**S.E**.	**Coeff**.	**S.E**.
Severity at baseline	-0.40***	0.08	-0.42***	0.07	-0.51***	0.07	-0.34***	0.07
Mean score improvement at week 2 vs. week 1	-6.16***	1.84	-4.62**	1.55	-8.52***	2.11	-4.24**	1.34
Mean score improvement at week 4 vs. week 1	-5.13***	1.84	-5.28***	1.55	-7.22***	2.11	-4.04**	1.34
Age	0.00	0.24	0.21	0.20	0.33	0.23	0.39*	0.18
Gender (female vs. male)	-15.74	19.73	3.83	16.67	2.18	19.38	13.26	15.06
Body Mass Index (<25 vs. >= 25)	-0.68	3.44	-1.03	2.92	-1.25	3.44	-1.19	2.65
Rescue drug (used vs. not used)	6.03	3.38	4.27	2.85	3.79	3.65	4.86	2.52
Aversion to cold (mild vs. moderate or severe)^c^	3.86	3.94	7.59	3.31	8.12*	3.88	3.93	3.09
Flaccidity of lower back/knee (mild vs. moderate or severe) ^c^	3.03	3.46	2.03	2.87	-1.36	3.34	0.65	2.63

^a^VAS = visual analogue scale as the dependent variables.

^b^Refers to the three subscales of the Western Ontario and McMaster Universities Osteoarthritis (WOMAC) Index as the dependent variables.

^c^Pattern identification as the independent variables.

*indicates *P *< 0.05; ** indicates *P *< 0.01; and *** indicates *P *< 0.001.

### Safety Issue

There were no significant changes in all tested biomarkers including the highly sensitive indicators of nephrotoxicity [23,24] after the 4-week *DJT *intervention. Only four adverse drug reactions were potentially related to *DJT *treatment, involving single events of skin discoloration, flashes, diarrhea and tachycardia.

## Discussion

This study is observational in nature and lacks a randomized placebo group. We, therefore, cannot make strong inference from the outcomes reported by the patients. However, the present study recruited only the patients with radiographically-verified OA of the knee and used self-comparison to rule out the potential confounders of the BMI, age, exercise and socioeconomic status [25]. This study also showed that the supposed efficacy of *DJT *on flaccidity of the lower back and knee and aversion to cold, as indicated in *Qianjin Yaofang *(*Invaluable Prescriptions for Ready Reference*) [4], were not reflected in the 'relief of symptoms' or 'physical functioning' scores for OA of the knee (Table 4).

In current practice of Chinese medicine, *DJT *is usually prescribed as a treatment for a combination of symptoms of pain, stiffness, flaccidity and/or aversion to cold. The results of this study suggest that the 4-week treatment of the *DJT *preparation may not be effective in treating flaccidity and/or aversion to cold. As most participants in this study experienced only mild or moderate symptoms of flaccidity and/or aversion to cold, very limited room may have been left for improvements of the symptoms (Table 2).

Following the recommendations of the World Health Organization, we used the WHOQOL-BREF, a multi-dimensional measure of QOL, as our secondary outcome in this study [26]. However, there were no statistically significant changes in the scores of either the WHOQOL domains or the different facets (Table 3) for those patients suffering from pain or stiffness who showed significant improvements. This may be due to the small sample size and the generic nature of the WHOQOL-BREF which, for most of the OA patients in this study with only mild symptoms, may not be so responsive.

Since *DJT *contains *Xixin *which may contain AA-I, an undetectable level of AA-I in the medications of the study was assured in compliance with the regulations of the CCMP. In contrast to the positive control sample directly taken from *Xixin*, no AA-I was detected in the independent chemical analysis of *DJT *by LC/MS/MS in this study.

The *DJT *dosage used in this study (2.5 g twice daily) is smaller than that in a previous study which showed that an oral daily dose of 9 g of *DJT *had a similar toxicity profile to that of diclofenic [11]. The results of this study provide (about IIb level [26]) that the participants seemed well tolerant of the 5.0 g daily dose of the *DJT *preparation for four weeks, and that the four adverse drug reactions noted earlier were not serious enough to have caused any withdrawal by any of the participants.

It should be noted that the results from the current study may not represent those of other types of *DJT *preparations, as different manufacturing processes may affect the biological activities and toxicity levels of herbs such as *Xixin*. Moreover, as the participants in this study were only Taiwanese patients suffering from OA of the knee during late spring and early autumn, we should remain cautious about generalizing these findings to cover patients in other settings, or indeed, to different racial groups.

## Conclusion

The participants indicated symptomatic improvements in pain, stiffness and physical functioning as demonstrated in the WOMAC subscale scores which began to decrease after two weeks of treatment. Multiple regression analyses, however, showed that the *DJT *preparation was less effective on flaccidity of the lower back and knees and aversion to cold. The dosage form and prescription pattern of the *DJT *preparation described in the current study may be used as a complementary or alternative treatment of pain, stiffness and other physical functioning problems in patients with OA of the knee. As the results from the current study could have been confounded by placebo effect, natural fluctuation, insufficient follow-up time and/or other unobserved factors, a prospective randomized, double-blind, controlled trial to further evaluate the efficacy of *DJT *on OA patients is warranted in future studies.

## List of abbreviations

DJT: Duhuo Jisheng Tang

WOMAC index: Western Ontario and McMaster Universities Osteoarthritis index

WHOQOL: World Health Organization Quality of Life

VAS: Visual analogue scale

OA: Osteoarthritis

AA-I: Aristolochic acid I

CCMP: Committee of Chinese Medicine and Pharmacy

GMP: Good Manufacturing Practices

HPLC: High performance liquid chromatography

LC/MS/MS: Liquid chromatography – tandem mass spectrometry

BUN: Blood urea nitrogen

AST: Serum aspartate-aminotransferase

ALT: Alanine-aminotransferase

GCP: Good Clinical Practices

NSAIDs: Non-steroidal anti-inflammatory drugs

WHOQOL-BREF: World Health Organization Quality of Life – brief version

QOL: Quality of life

A/G: Albumin/globulin

BMI: Body mass index

## Competing interests

The author(s) declare that they have no competing interests.

## Authors' contributions

JNL did the study design, patient recruitment, manuscript preparation and submission. HJC and CCC did the patient recruitment. JSH did all statistical analyses and interpretation of data. JHL did the LC/MS/MS analysis. JDW conceived the study, did its design and coordination, and helped draft the manuscript. All authors have read and approved the final manuscript.
